# Low-Carbon Concrete Based on Binary Biomass Ash–Silica Fume Binder to Produce Eco-Friendly Paving Blocks

**DOI:** 10.3390/ma13071534

**Published:** 2020-03-27

**Authors:** André Henrique Campos Teixeira, Paulo Roberto Ribeiro Soares Junior, Thiago Henrique Silva, Richard Rodrigues Barreto, Augusto Cesar da Silva Bezerra

**Affiliations:** 1 Department of Transports Engineering, Federal Centre for Technological Education of Minas Gerais, Belo Horizonte 30421-169, Brasil; andrehenrique@cefetmg.br; 2 Department of Materials Engineering, Federal Centre for Technological Education of Minas Gerais, Belo Horizonte 30421-169, MG, Brazil; pauloroberto.rsoares@gmail.com (P.R.R.S.J.); silva.thenrique@gmail.com (T.H.S.); richbarrto@gmail.com (R.R.B.)

**Keywords:** low-carbon concrete, waste binary binder, alkali-activated material, biomass ash, silica fume, eco-friendly paving block

## Abstract

The civil construction industry consumes huge amounts of raw materials and energy, especially infrastructure. Thus, the use of eco-friendly materials is indispensable to promote sustainable development. In this context, the present work investigated low-carbon concrete to produce eco-friendly paving blocks. The binder was defined according to two approaches. In the first, a binary binder developed with eucalyptus biomass ash (EBA) and silica fume (SF) was used, in total replacement for Portland cement. In the second, the mixture of residues was used as a precursor in alkali-activation reactions, forming alkali-activated binder. The experimental approach was carried out using five different mixtures, obtained by varying the amount of water or sodium hydroxide solution. The characterization of this new material was carried out using compressive strength, expandability, water absorption, deep abrasion, microstructural investigation, and organic matter degradation potential. The results showed that the EBA-SF system has a performance compatible with Portland cement when used as an alternative binder, in addition to functioning as a precursor to alkali-activated concrete. The blocks produced degraded organic matter, and this degradation is more intense with the incidence of UV. In this way, the EBA-SF binder can be successfully used for the manufacture of ecological paving blocks with low carbon emissions.

## 1. Introduction

The construction industry consumes a large number of resources and energy, in contrast to other sectors of the contemporary economy [1]. In this scenario, concrete stands out as a construction material that is used extensively in infrastructure and building works [2]. Consistent data presented by the UN Environment et al. [3] revealed that cement is the most used material in the world when compared to other materials such as wood, iron, ceramics, and asphalt. Considering that cement represents only 15% of concrete (by mass), the impact generated by construction activities is evident [4]. This massive demand for inputs requires, necessarily, care with the environment. The United Nations Organization, through the 2030 Agenda [5], defined several objectives to promote sustainable global development, among which objectives 9, 11, and 13, related to infrastructure, sustainable cities, and change, stand out in the global climate, respectively. The fulfillment of the mentioned objectives is inevitably achieved by the adoption of sustainable practices in the construction sector [6]. The possibility of using waste to replace traditional Portland cement-based building materials promotes the mitigation of environmental impacts. It gives civil construction a convergent character with global sustainable development trends [7,8,9,10].

Aiming at the growing demand for ecological materials, supplementary cementitious materials (SCM) appear as an alternative to traditional Portland cement [11,12]. Generally, in the form of industrial and agricultural waste, such as silica fume, blast furnace slag, fly ash, rice husk ash, and sugar cane bagasse ash, SCM are successfully used for the production of green binders with low emissions carbon [13,14,15,16,17,18]. In addition to environmental advantages, such as reduced CO_2_ emissions, reduced energy expenditure and sustainable destination for solid waste, the use of SCM often implies performance improvements in the resulting cementitious composite [19,20]. This optimized performance can be achieved in terms of mechanical behaviour (compressive strength, bending, etc.), or related to parameters that indicate durability (porosity, resistance to chloride penetration, resistance to carbonation, accelerated aging, etc.) [21,22,23,24]. As the knowledge on the subject advances, the percentage of substitution tends to increase, and new mixtures of Portland cement with SCM are developed. The relevant literature emphasizes partial replacements, which have a wide range of coverage (3–80% SCM), resulting in binary, ternary, or quaternary binders, depending on the supplementary materials used [25,26,27,28,29,30]. However, total replacement (100% SCM) needs to be further investigated [31]. Thus, to expand the possibilities of using SCM, to contribute even more to the solution of environmental problems and to boost sustainable development, the present work evaluated total substitutions of Portland cement by SCM. 

Biomass ash (BA) is an advantageous option for use in eco-friendly blended cement [13,23,32,33,34]. Obtained by burning waste in electric power plants, BA can originate from any organic material, mainly energy crops destined to get biomass and energy production such as oilseed crops (coconut, sunflower, olives, etc.), starch crops and sugar (sugar beet, sugar cane, etc.), lignocellulosic crops (grass and wood, including eucalyptus) or solid residues from agriculture, the food industry, the wood industry, animal husbandry, and various industrial processes (remains of crops, bagasse, straw, shavings, sawdust, biodegradable animal and wastewater treatment waste, etc.) [35,36,37]. Several kinds of research have been carried out to enable the use of BA in cement and concrete [38,39,40]. Currently, tons of ash is generated by burning wood in the form of firewood, residues, or small pieces processed (chips). As it is a renewable energy source, the trend is that the consumption of wood, mainly eucalyptus, will increase substantially in the coming years, generating vast amounts of ash. As supplementary cementitious materials, we highlight the ash from burning wood, which is called wood biomass ash (WBA) or wood ash (WA) [13]. In general, WBA works as a pozzolanic material [41], promotes a filler effect [42], can be used as a binder with other SCM [43], or acts as a precursor to alkali-activated materials [44,45]. The properties that are conferred to cementitious materials with WBA range from optimized mechanical behavior [46,47,48] to improvements in term and durability [49,50].

Silica fume (SF) is a by-product from the production of metallic silicon and ferrosilicon alloys [51]. In the form of a very fine powder, it has tiny spherical particles, a high content of amorphous silica, high pozzolanic activity, and high reactivity [11,29]. In general, SF can be used to replace Portland cement partially, as an addition, promoting significant performance improvements, both in the fresh and hardened state [51,52]. In addition, it can be used as a precursor in alkali-activated materials, which are often associated with other SCM [53]. The addition of SF promotes the densification of the cement matrix and makes the interfacial transition zone more cohesive, improving the bond between paste and aggregates. Consequently, the mechanical behavior is improved, with a significant increase in flexion, traction, and compressive strength [54,55,56]. In terms of durability, the use of SF decreases permeability and increases resistance to corrosion, attack by sulfates, and surface wear [57,58,59,60]. For this reason, SF is widely used in high-performance concretes and has become an SCM with high added value [61,62,63].

In line with eco-friendly materials, several kinds of research are carried out on alternative binders, such as calcium aluminate cement, calcium sulfoaluminate cement, super sulfated cement, and alkali-activated materials (AAM) [64,65,66,67]. AAMs are formed from the alkali-activation reaction, in which a specific type of solid aluminosilicate reacts in an aqueous alkaline medium, producing a binder composed of hydrated alkali-aluminosilicate phases. According to the definitions in the recent literature, precursors are sources of aluminosilicates, while activators induce alkaline conditions [68,69]. In addition, research shows that materials produced with activated alkali binders can achieve mechanical properties and durability similar or superior to cementitious materials [70,71,72,73]. AAMs have gained prominence in the scientific community, since they are generally produced with industrial waste also, creating an ecological and sustainable alternative to traditional building materials [74,75,76].

Functional materials are at the forefront of material research because of society’s growing technological advancement and the need for increasingly specific applications [77,78]. In the field of construction materials, these applications are often aimed at preserving the environment, such as effluent decontamination, CO_2_ capture and degradation, self-cleaning surfaces, and cleansing materials, among others [79,80,81,82]. The depolluting and self-cleaning properties are related to the photocatalytic potential of the material and the physical surface phenomena. The material acts as a catalyst and site for reactions of oxidation and degradation of organic matter, while the surface becomes super-hydrophilic or super-hydrophobic [79,80,83]. However, most studies on photocatalysis in construction materials are based on the photocatalytic effects of titanium oxide (TiO_2_), which is often nanostructured in thin films, added to coatings such as paints and sealants or mixed in cementitious materials [84,85]. These processes are financially expensive. Therefore, natural materials with photocatalytic, self-cleaning, and non-polluting properties are viable alternatives to spread functional construction materials.

On the other hand, infrastructure works serve as a basis for promoting world growth and improving people’s quality of life [86]. Without the necessary infrastructure and facilities, it is not possible to move or transport cargo (roads, railways, ports, airports, etc.), obtain treated water (drinking water supply system), discharge and treat effluents in suitable locations (sewage system) sanitation), have access to services (lighting, energy, gas, telephony, internet, etc.), or maintain the functioning of industries and agriculture [87,88,89]. Objective 6, “clean water and sanitation”, and objective 9, “industry, innovation and infrastructure”, of the United Nations Agenda 2030 [5] show that global sustainable development is closely associated with the infrastructure necessary to meet the demands of each sector of contemporary society. Paving works are essential to the basic infrastructure. Due to the relevance of this type of construction, which has (i) a high consumption of raw materials, (ii) large extensions of the built environment (usually concrete or asphalt), (iii) permanent contact with the external environment, and (iv) continuous traffic vehicle and people, new eco-friendly floors have emerged to meet the demand for sustainable materials [90,91,92,93,94,95,96,97,98]. In this scope, interlocking pavements (paving blocks or paver) are widely used, and their non-structural application increases the use of waste as raw materials (aggregates, replacements, etc.) without the need for excessive requirements with high performance, but meeting the specifications required project [99,100,101,102,103]. Given the characteristics mentioned above, the use of floors with photocatalytic properties and the consequent self-cleaning capacity boosts the preservation of the environment [104].

Another important aspect is that cities are increasingly impermeable, especially in developing countries. This waterproofing is mostly due to the intensive use of waterproof asphalt and concrete paving, which in addition to significantly reducing the infiltration of rainwater into the soil also increases the speed of its accumulation. Impermeabilization of the land combined with disorganized and uncontrolled urban growth or even urbanization in basins more prone to intense rainfall impacts with reduced infiltration and the absence of natural defences leads to incidents such as floods. Flash floods are among the most catastrophic natural hazards that disrupt the environment and societies [105], besides being the most expensive form of natural disaster [106], especially when linked to watercourses such as rivers [107]. Floods in urban areas cause significant damage to infrastructure, communities, and the environment [108]. Recent episodes of floods in cities in Brazil [109,110,111,112,113,114], for example, could be minimized with more appropriate urban occupation and urban green infrastructure, such as innovative ’green street’ techniques, permeable pavements, green walls, or rain gardens [115]. In this sense, concrete block floors are more permeable than conventional asphalt or Portland cement concrete floors.

Given the above, there is an intense movement by the scientific community to promote new discoveries related to eco-friendly materials, with a view to sustainable development. When it comes to infrastructure works, the challenges are accentuated by the large volumes of raw material used. The use of waste as a partial replacement for Portland cement is addressed in several works in the relevant literature [3,57,116,117,118,119,120,121,122]. Other research investigates the development of alkali-activated materials from supplementary cementitious materials [123,124]. However, a joint approach on total substitution of Portland cement, alkali-activated binders, and the use of waste as raw material, with a focus on the production of pavers, is still poorly available in the literature. In this scenario, the present work studied low carbon concrete, using binary binder composed of biomass ash and silica fume, to produce eco-friendly paving blocks. The characterization of concrete pavers was carried out comprehensively, from strength to microstructural investigation. In addition to the low carbon emission associated with the use of waste, the potential for degradation of organic matter and consequent self-cleaning properties of the produced concrete was investigated. Combining the use of waste with self-cleaning properties, without the addition of photocatalytic oxides or additional processing, provides a functional material with high relevance to the environment, in addition to reducing production costs.

## 2. Experimental Study

### 2.1. Materials and Mix Proportion

**Figure 1:**
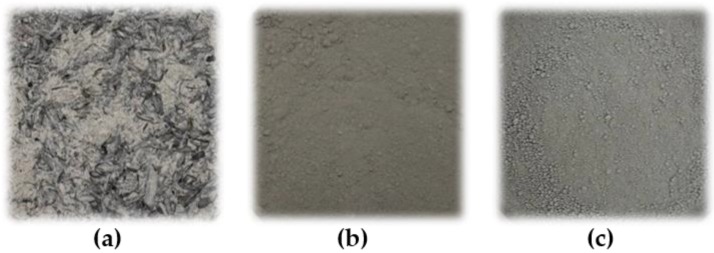
Waste used to replace Portland cement (**a**) eucalyptus biomass ash (EBA) before grinding (**b**) EBA after grinding and (**c**) silica fume.

The selection of materials was carried out to prioritize eco-friendly inputs. Eucalyptus biomass ash (EBA) comes from a thermoelectric plant, while fume silica comes from the metal silicon manufacturing industry. Both by-products were generated in Brazilian industries. The EBA in nature (Figure 1a) was processed using grinding to disaggregate the material and homogenize the grain size, using a high-performance planetary mill. After the comminution process, EBA has a powdery appearance and dark gray color, similar to Portland cement (Figure 1b). The active silica has a homogeneous fine powder aspect and a light gray color tone. As it is a very thin material, the presence of agglomerated particles is easily seen (Figure 1c). The chemical composition of the EBA and SF were determined by the lithium tetraborate fusion method and quantification in an energy dispersive X-ray fluorescence spectrometer (XRF) Panalytical Axios Fast (Table 1). EBA has a high calcium oxide content (44.71%) and a high loss on ignition (30.22%). SF has a high content of silicon oxide (95.41%) and low loss on ignition (0.34%).

**Figure 2:**
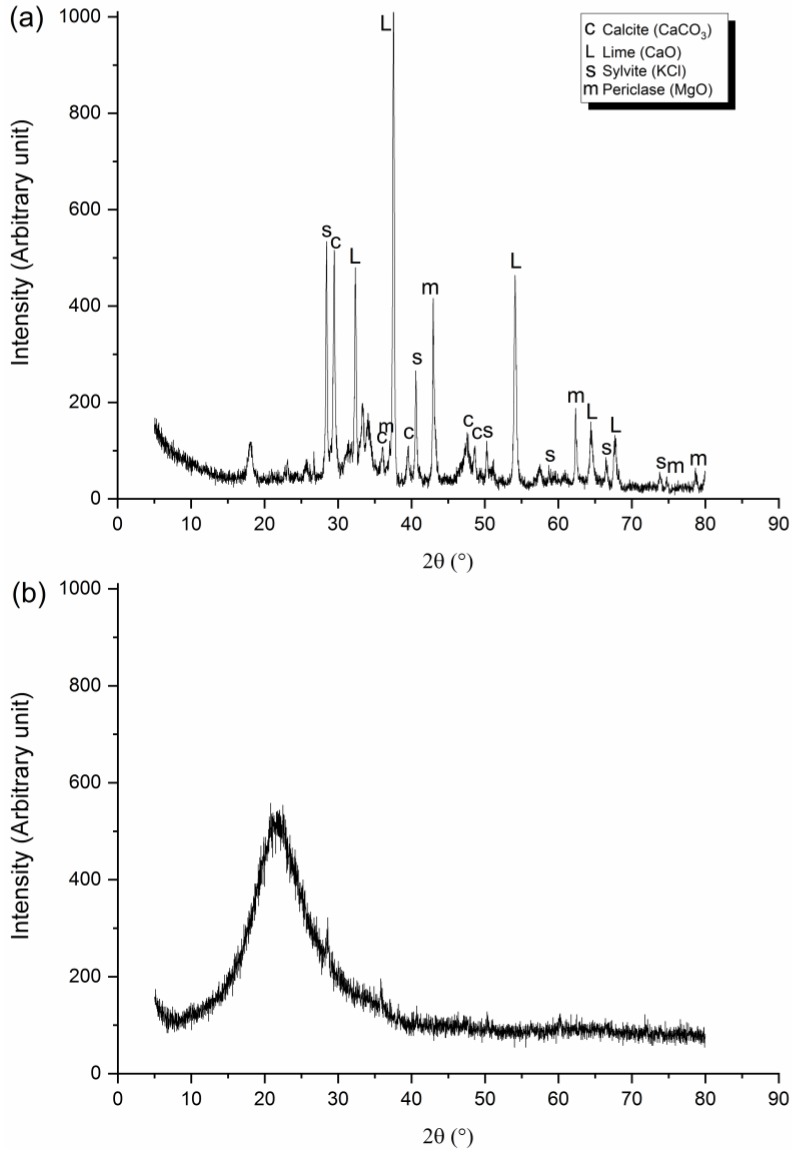
XRD of (**a**) EBA and (**b**) SF.

The crystalline phases in the ash and resultant binders were identified by using X-ray diffraction; a Shimadzu XRD-7000 (Kyoto, Japan) was used, with a copper X-ray tube operated at 40.0 kV and 30.0 mA, with a 0.020° 2θ sweep per step, from 5° to 80° at 0.6 s/step. For this, pastes (without aggregates) of the BBC06, BBC05, and AABC06 dosages were produced. MATCH! software was used to evaluate the crystalline phases of the pastes (Version 3.7.0.124) using the powder diffraction method and using Crystallography Open Database (COD). EBA and the SF had their phases identified and presented in Figure 2a,b, respectively. In the EBA, peaks of the calcite (COD 9007689), lime (COD 1011327), sylvite (COD 9003130), and periclase (COD 9006456) phases were identified. In the SF, no crystalline phase peaks were identified.

**Figure 3:**
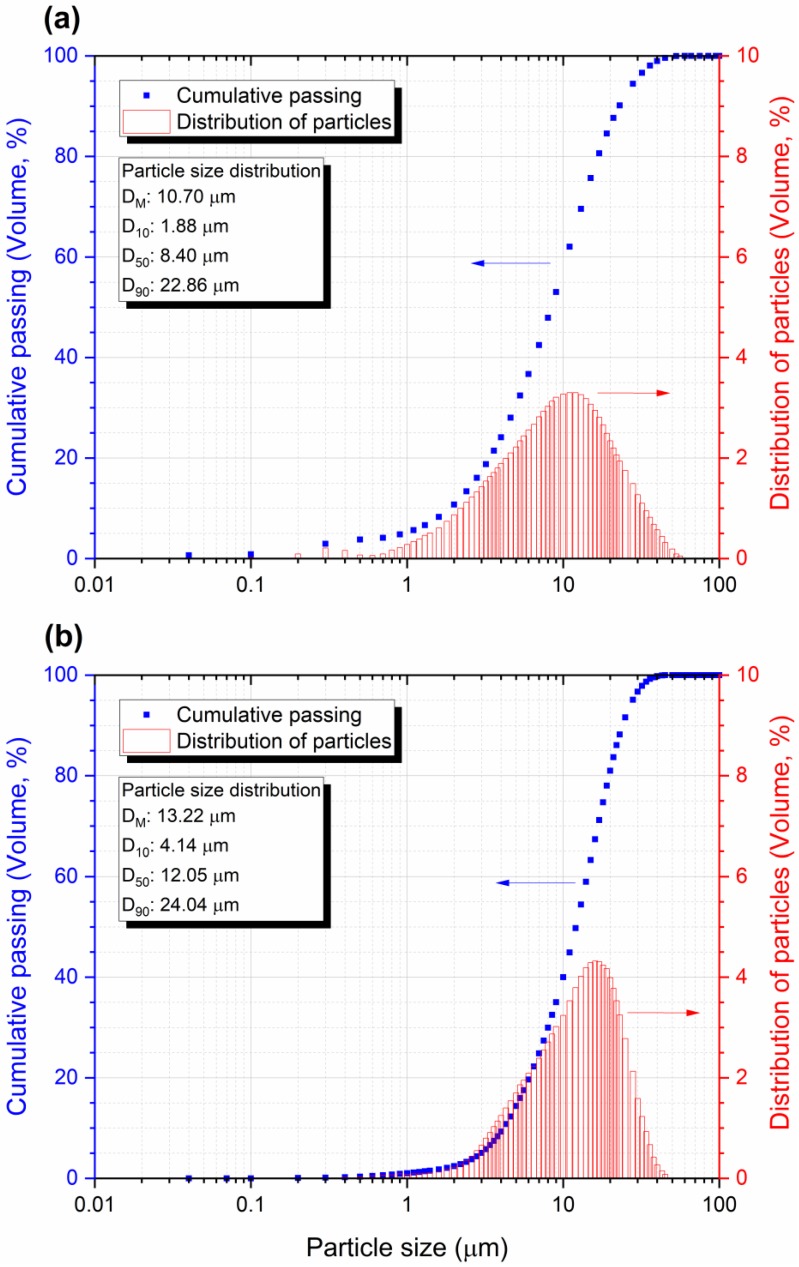
Particle size distribution curve with a histogram for (**a**) EBA and (**b**) SF.

**Figure 4:**
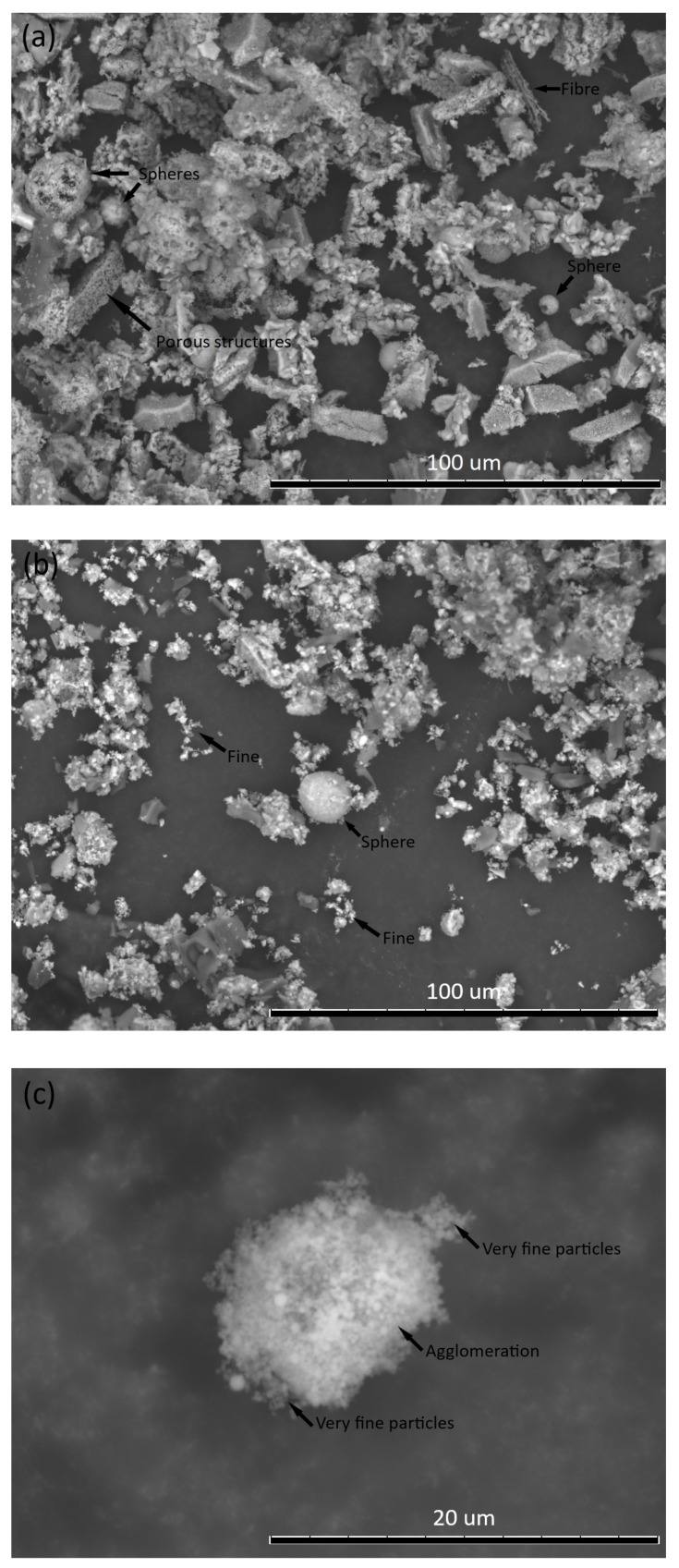
Images obtained by SEM (**a**) EBA as received, (**b**) ground EBA, and (**c**) SF.

The average particle size and particle size distribution of EBA and SF obtained by laser diffraction are very close (Figure 3). However, although the SF is slightly coarser, the SEM images show that the SF is much thinner than the EBA (Figure 4b,c). EBA before grinding processing presents spherical particles, some of which are massive, while others are hollow. It is also possible to observe porous structures and fibers with incomplete combustion (Figure 4a). EBA after grinding processing has significantly smaller and more fragmented particles than EBA before grinding. However, it is still possible to observe spherical particles (Figure 4b). The SF is extremely thin, which is consistent with the literature. However, the particles are agglomerated, which is what led to the presented particle size (Figure 4c).

**Figure 5:**
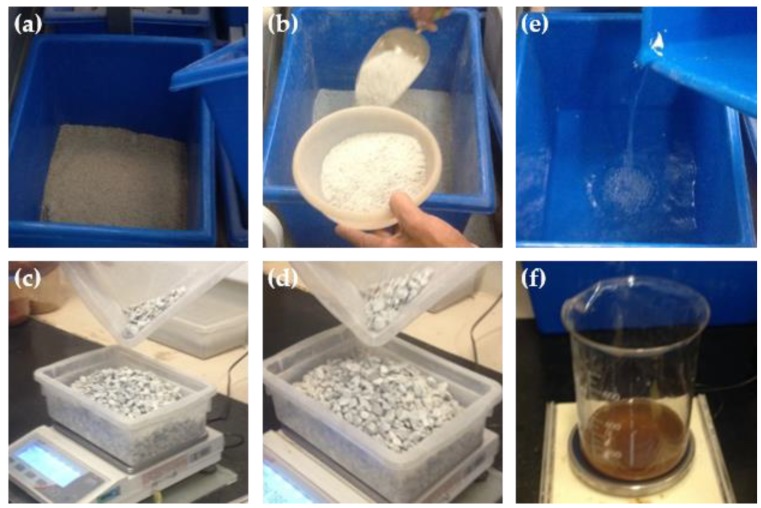
Inputs used in low-carbon concrete (**a**) fine sand, (**b**) stone powder, (**c**) and (**d**) coarse aggregate with dimensions of 4.8 to 9.5 mm and 9.5 to 19.5 mm respectively, (**e**) water and (**f**) superplasticizer additive.

As aggregates, fine sand (Figure 5a), stone powder (Figure 5b), and artificial gravel with two different granulometries (Figure 5c,d) were used. The workability of the concrete was corrected with the use of a superplasticizer additive (Figure 5e). The water was supplied by the local supply system (Figure 5f).

To provide as many results as possible for an alternative binder based on the combination of EBA and SF (called the binary EBA-SF system) and to expand the contribution of the present work, two approaches were carried out. The first concerns the use of binder with the total substitution for Portland cement. In this case, equal proportions of EBA-SF were used, and the mixture was made using water, generating a binary binder with 100% residues. In the second approach, the sodium hydroxide solution was used instead of water to form an alkali-activated binder, maintaining equal proportions of EBA-SF. Therefore, the alkaline solution acts as an activator, and the EBA-SF system acts as a precursor. The dosage of low-carbon concrete was eminently experimental. A total of five mixtures were defined, which are identified in Table 2, with three mixtures of concrete with binder without alkaline activation and two others with an alkali-activated binder. The materials were dosed to obtain a homogeneous mixture to provide the necessary performance for the manufacture of paving blocks. The traditional dosages of concrete with Portland cement provided by Mehta and Monteiro [125] and Neville [52] served as a basis for defining the proportions with alternative binders. The variables addressed in this study correspond to the influence of two factors: (i) whether to adopt an activating solution and (ii) the water/binder (w/b) or solution/binder (s/b) relationships. The first mixture (BBC-WCA: binary binder concrete-without coarse aggregate) was defined without coarse aggregate to assess the water demand for the waste. Then, two other mixtures were defined using the EBA-SF system and water (BBC06: binary binder concrete with w/b = 0.6 and BBC05: binary binder concrete with w/b = 0.5). In the last two mixtures, the water was replaced by sodium hydroxide solution with a concentration of 3 mol/L, producing an alkali-activated binder (AABC07: alkali-activated binder concrete with s/b = 0.6 and AABC07: alkali-activated binder concrete with s/b = 0.7). Although the dosage was experimental, the aggregates were chosen to make the particle dimensions in the concrete compatible. Thus, two types of fine aggregates (sand and stone powder) and coarse aggregates with two different particle sizes were used. Aggregates with different sizes complement each other and give concrete better particle packing [126].

In the context of alkali-activated materials, the properties of the precursor and the activator directly influence the structure and behavior of the binding matrix [69]. The chemical composition of the residues indicates that the EBA-SF mixture works as a mixed system of high calcium precursors, with EBA representing a source of calcium, while SF provides the silicates. The mixture of the two residues was designed to balance the availability of calcium and silicates. In general, the alkali-activation process of high calcium mixtures is heterogeneous and comprises, among other mechanisms, particle dissolution, nucleation, and solid-phase growth [127]. The characteristics of the residues, such as chemical composition and fine granulometry translated in terms of surface area and particle morphology, act synergistically, favoring the surface and interface mechanisms, and promoting conditions for alkali-activation reactions. Recent work has addressed the use of biomass ash as precursors to AAMs and obtained favorable results [44,45]. In turn, activators function as an alkaline medium to promote alkali-activation reactions [64]. We chose to use aqueous sodium hydroxide solution, which is commonly used as an activator for AAM. Activators based on sodium hydroxide have an excellent chemical affinity with high calcium precursors, efficiency in activating reactions, and a low viscosity of solutions, in addition to availability for obtaining [128]. Despite the pertinent literature indicating concentrations starting at 5 M [129], in the present study, the concentration of the activating solution was defined at 3 M; this figure is in agreement with previous research [130,131], in which consistent results were obtained for low molar concentrations.

**Figure 6:**
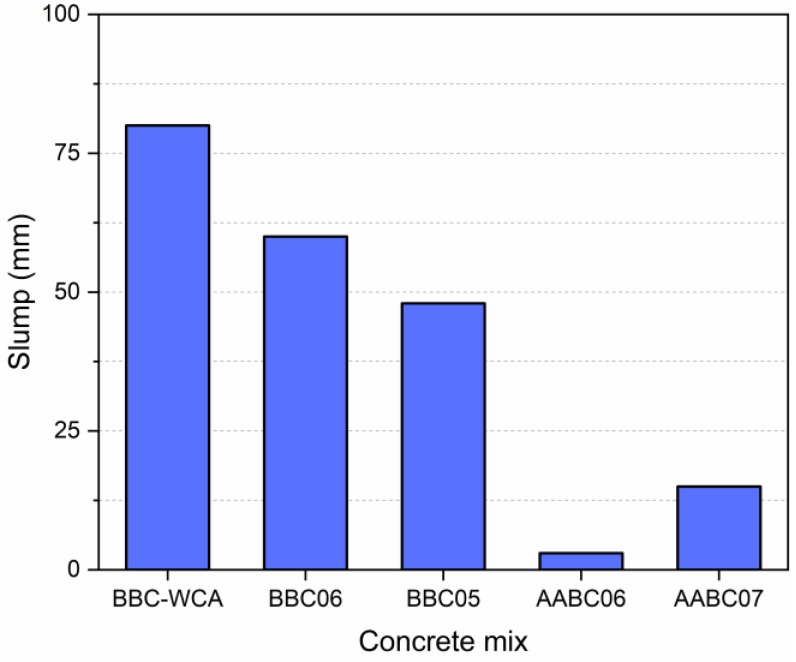
Consistency in the fresh state for each concrete mixture measured in terms of the slump test.

The consistency of fresh concrete can be related to workability and was used as a criterion to define the w/b and s/b ratios. The slump test was chosen to measure consistency. Figure 6 shows the slump test values for each mixture. When defining the BBC WCA mix, the slump was accentuated; that is, the w/b = 0.8 ratio can be reduced. Thus, we opted for the w/b = 0.6 (BBC06) ratio and followed by the w/b = 0.5 (BBC05) ratio. The alkali-activated concrete was initially dosed with s/b = 0.6. However, the slump was practically null, which led to an increase in the s/b factor. This indicates greater demand for water because of the reactions relevant to alkali activation. This methodology aimed to evaluate an optimum point of w/b or s/b so that the amount of water or solution would be ideal for concrete-hardening reactions.

### 2.2. Preparation of Concrete Paving Blocks Samples

**Figure 7:**
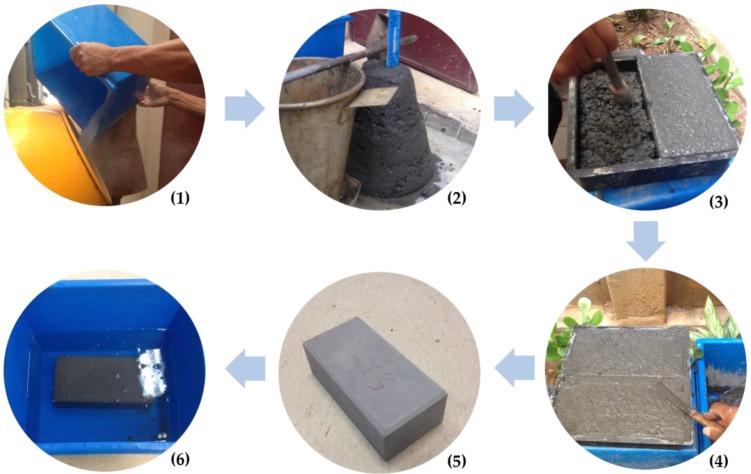
Production steps for concrete paving blocks (**1**) mixing of materials, (**2**) slump test, (**3**) molding and thickening, (**4**) surface finishing, (**5**) blocks after de-molding, and (**6**) submerged curing in water.

The materials were mixed using a mechanical mixer for concrete (Figure 7-(1)). First, the mixer drum was moistened with water, so that there was no material adherence and moisture capture. The superplasticizer additive was diluted in the water fraction for better distribution over the volume of material. Then, half the volume of water with superplasticizer was added, together with the coarse aggregates. The mixer was turned on for 30 s for pre-mixing. Then, the other materials (fine aggregates and EBA-SF residues) and the remaining water with superplasticizer were added. The mixing was carried out until the concrete obtained a uniform appearance. In the case of the production of concrete with activated alkali binder, the executive process followed the same steps, but instead of water, sodium hydroxide solution was used.

After the mixing was completed, the concrete was subjected to the slump test (Figure 7-(2)) to evaluate the consistency and workability. The molding of the blocks (Figure 7-(3)) was made using plastic shapes with dimensions of 25 cm × 12.5 cm × 8 cm. During molding, manual concrete densification was carried out, with two overlapping layers. It was necessary to finish the surface with the help of a spatula (Figure 7-(4)) to level the faces of the block and promote the correct stress distribution during mechanical testing. After 24 h of hardening under controlled humidity and temperature, the blocks were de-molded and presented a well-finished appearance, with a flat and smooth surface (Figure 7-(5)). Right after de-molding, the blocks were inserted in water for submerged curing for 28 days (Figure 7-(6)). The complete mixing, moulding, and curing process was carried out at a temperature of 24 ± 2 °C and relative humidity of 60% ± 5%.

### 2.3. Performance Evaluation

The performance evaluation of low-carbon concretes was carried out using the following approaches, all after 28 days of curing: (i) mechanical behavior, (ii) water absorption, specific mass and porosity, (iii) dimensional variation, (iv) abrasion resistance, (v) microstructural investigation, and (vi) potential for organic dye degradation.

The mechanical behavior was evaluated at 28 days for all mixtures, using the paving block compression test. The test was performed in universal test equipment with controlled load application and measurement using a 300 kN load cell. The maximum loads were measured, and the compression stresses were calculated according to the area of application of the efforts. Thus, the compressive strength values for concrete blocks measured in MPa were obtained. Four specimens of each trace were used, and the average values were calculated, together with the respective standard deviations.

In terms of physical properties, water absorption (WA), porosity (PR), dry density (ρdry), and wet density (ρwet) were evaluated. All properties were evaluated using the water absorption test. In this test, the mass of the blocks was evaluated in different situations. After curing for 28 days, the blocks were removed from water immersion, the surface was dried, and the first weighing was performed, obtaining the saturated mass (M_sat_). Then, the submerged weight of the blocks was evaluated, using a hydrostatic balance, resulting in the submerged mass (M_sub_). Finally, the blocks were placed in the greenhouse for 24 h until mass consistency at 105 °C, and then the dry mass (M_dry_) was obtained. The calculation of physical properties was performed using Equations (1), (2), (3), and (4), with ρw = 1.00 g/cm^3^ being the water density:

(1) $$\mathrm{WA}\;(\%)=\frac{M_{sat}-M_{dry}}{M_{dry}}\times 100$$

(2) $$\mathrm{PR}\;(\%)=\frac{M_{sat}-M_{dry}}{M_{sat}-M_{sub}}\times 100$$

(3) $$\rho_{\mathrm{dry}}\;(g/{\mathrm{cm}}^{3})=\frac{M_{dry}}{M_{sat}-M_{sub}}\times \rho_{w}$$

(4) $$\rho_{\mathrm{wet}}\;(g/{\mathrm{cm}}^{3})=\frac{M_{sat}}{M_{sat}-M_{sub}}\times \rho_{w}$$

The dimensional stability of the blocks was evaluated using the expandability test, in which the concrete blocks were subjected to wetting and drying cycles, and the dimensions between the middle thirds of the most extensive face were obtained. During drying, the blocks were placed in an oven at a temperature of 105 ° C for 24 h. After removing from the greenhouse, the concrete blocks were left in the air until reaching stabilization at room temperature. Wetting was performed by complete immersion in water for 24 h. Then, the blocks were removed from the immersion, and the surface was dried with the aid of a cloth. The distance between the middle thirds was measured both after drying and wetting, always at room temperature.

The abrasion resistance was evaluated using the deep abrasion test, which simulates traffic conditions on the concrete blocks and evaluates the surface wear by friction. In this test, the concrete block is inserted in rotating support equipment, using sand as an abrasive material. The block is compressed against the support, which promotes continuous wear. The test lasted 2 h, with angular velocity adjusted to 15 revolutions per minute, corresponding to a course of 1130 m.

The microstructural investigation was carried out using an electronic scanning microscope (SEM) of the Hitachi brand, model TM3000. The equipment has a low vacuum and a backscattered electron detector (BSE). The images were taken with a voltage of 15 kV. To make the images, small fragments of the blocks were extracted using a cutting machine. The samples were taken under a microscope without additional preparation and fixed with high conductivity carbon tape.

**Figure 8:**
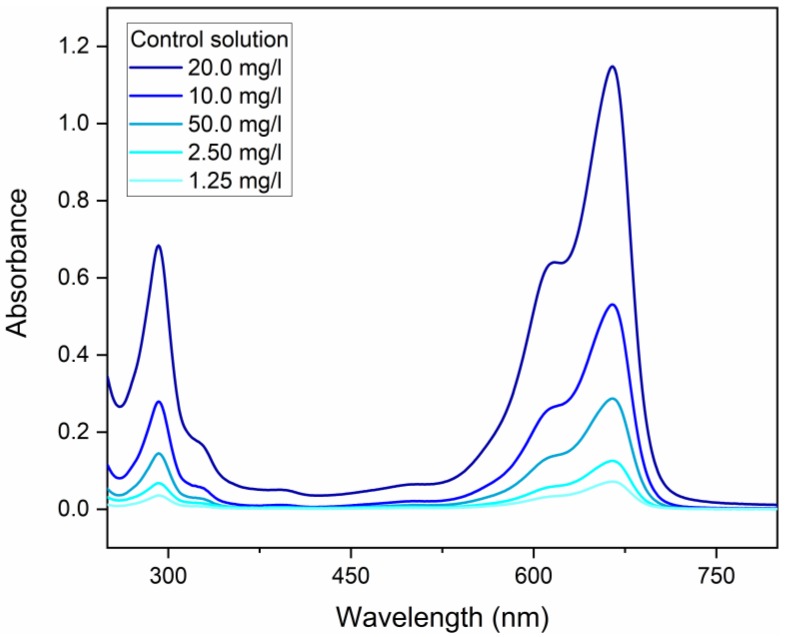
Absorbance of solutions in different concentrations.

**Figure 9:**
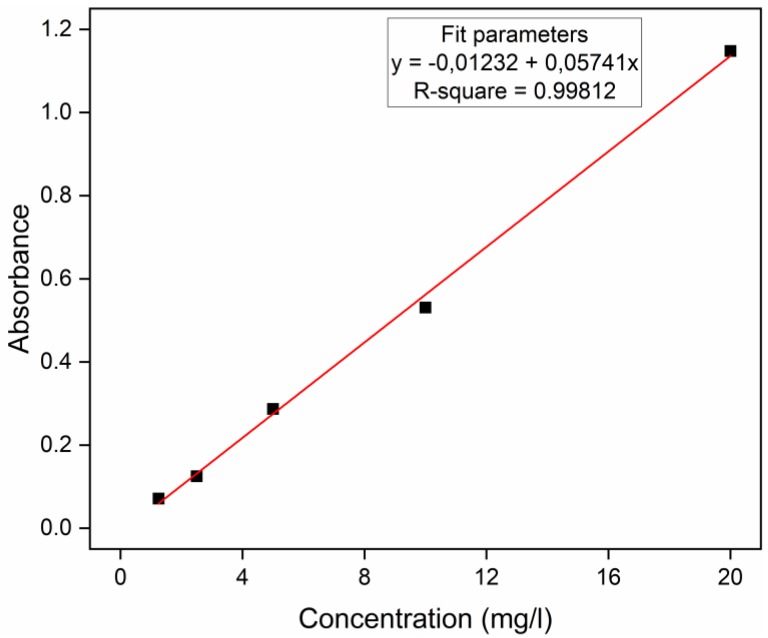
Calibration curve of absorbance versus concentration.

A property of great importance for new binders would be the ability to degrade organic matter, either to degrade organic dyes in wastewater or even to produce self-cleaning facades [132]. For the evaluation of the organic matter (pollutants) degradation potential, methylene blue (MB) compound was used [133]. The potential for organic matter degradation by eco-friendly concrete samples was realized with and without the incidence of ultraviolet (UV) light. Cube mortar specimens (sawn of eco-friendly paving blocks) were in 200 mL of distilled water solution with 20 mg/L MB. The mean mass of the samples was 47.85 g ± 1.72 g. The MB degradation was evaluated under two lighting conditions for the same specimens, with the first 24 h of degradation in a dark room (no light); at the end of this first cycle, the MB solution was replaced by a new solution of the same initial concentration, and the specimens were placed for a further 24 h in a chamber with 390 mm × 600 mm × 400 mm internal dimensions and two G15T8 UV-C 15 W lamps operated at 55 V, with 450 mm length and 254 nm UV-C emission and UV power equal to 49 uW/cm^2^ at 1 m. An aliquot of 10 mL of solution was collected at the beginning of the test and then at 2, 4, 8 and 24 h of each cycle, and absorbance measurements were performed from 250 to 850 nm in a Lambda 1050 UV/Vis Spectrophotometer. The calibration curve was constructed with 1.25, 2.50, 5.00, 10.00, and 20.00 mg/L concentrations, and the 664 nm peak was used for quantification. In Figure 8, it is possible to observe the UV-vis adsorption curves of the MB solutions (Pollutant) with 1.25, 2.50, 5.00, 10.00, and 20.00 mg/L concentrations. The existence of two different absorbance peaks at wavelengths equal to 292 nm and 664 nm can also be appreciated. To evaluate the concentration of the MB solutions collected during the tests, a calibration curve was created from the absorbance at the wavelength 664 nm of the solutions in the different concentrations (Figure 9).

In both steps, a control container containing only the AM solution was added to the UV chamber to assess the degradation of the dye without the influence of the catalyst material. Thus, if the MB concentration of the solutions with the samples of the eco-friendly paving blocks exposed to UV for 24 h is lower than the MB concentration of the solutions with the samples of the eco-friendly paving blocks without exposure to UV after 24 h indicates a possible self-cleaning property under UV action.

## 3. Results and Discussion

### 3.1. Compressive Strength

**Figure 10:**
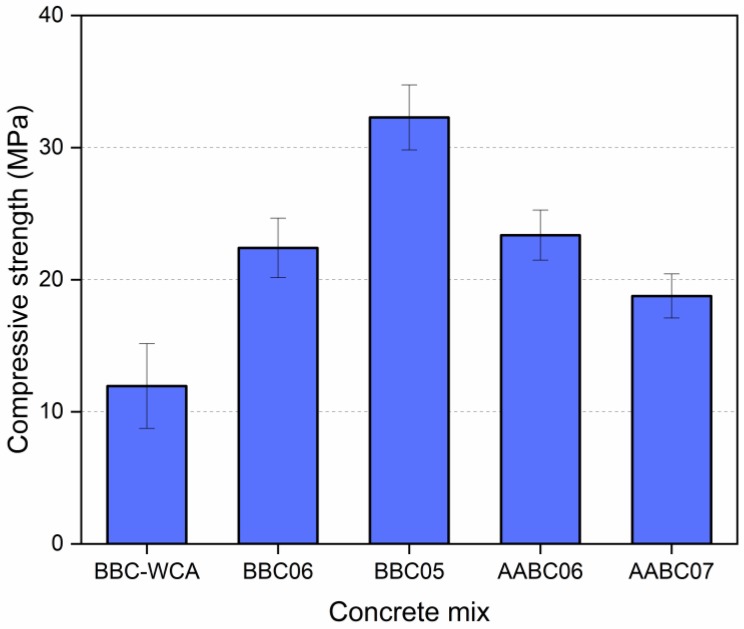
Compressive strength for each concrete mix.

In terms of mechanical behavior, it is observed that all mixtures reached representative levels of compressive strength (Figure 10). The lowest strength was achieved by the BBC-WCA mixture (11.97 MPa), while the highest was achieved by the BBC05 mixture (32.28 MPa). The other mixtures BBC06, AABC06, and AABC07 resulted in intermediate strength of 22.41 MPa, 23.39 MPa, and 18.77 MPa, respectively. In general, the values are in line with other studies [90,93,101], and the mixes BBC05, BBC06 and AABC06 meet the recommendations of the ASTM C902-15 [134] standard for paving blocks for internal and external use, both with minimum compressive strength of 20.70 MPa. This behavior shows that there was significant hardening and strength gain at 28 days. For all mixtures, the EBA-SF waste system functioned as a binding and cementing material, with a dense matrix that agglutinated the aggregate particles. In mixtures with binary binder and 100% substitution (BBC-WAC, BBC-06, and BBC05), water solubilizes the EBA-SF system and the reactions between the components result in the formation of solid products. Therefore, the EBA-SF system functioned as an alternative hydraulic binder in total substitution to Portland cement. On the other hand, when using calcium hydroxide solution, alkaline hydrolysis reactions and the formation of a hydrate system occur. In this case, the EBA-SF system works as a precursor, while the alkaline solution works as an activator, forming concrete with an alkali-activated binder.

Despite the strength gain, there was a significant difference between all evaluated mixtures. The low strength of concrete with binary agglomerate and without coarse aggregates (BBC-WCA) can be explained by the absence of coarse aggregates and the water-agglomerating factor (w/b). Coarse aggregates act in the distribution of stresses over the volume of material and act as a barrier against the propagation of microcracks [135]. Therefore, the absence of coarse aggregates caused a drop in strength. The water/binder ratio (w/b) played an influential role in mechanical behavior. As mentioned in the “Experimental Study” topic, the BBC-WCA mixture was a reference for adjusting the w/b value to assess the water demand for the waste. For this reason, a higher value was chosen (w/b = 0.8) when compared to other dosages common in the literature [125]. The water used in mixing the concrete components fluidizes the system and promotes sliding between particles, resulting in workability. If there is more water, the system becomes more fluid, and the workability increases, which was observed by the more significant slump of the BBC-WCA mixture observed in Figure 6. However, the excess water content increases the pore network of the material and consequent fall of strength. So, there was an excess amount of water due to the lower strength.

The other four mixtures evaluated have coarse aggregates of the same type, which promotes the correct distribution of stresses and removes the effect associated with the absence of coarse aggregates. Therefore, there is a significant strength gain when compared to the BBC-WCA mixture. The variables under analysis were (i) the influence of alkali activation and (ii) water/binder or solution/binder relationship. At first glance, alkali activation generated less meaningful results in terms of compressive strength. However, the amount of water used in the mixture must also be evaluated to have a set of conditions that make such comparisons feasible. The BBC06 and AABC06 mixtures have the same water content as a function of the binder (w/b = 0.6 and s/b = 0.6) and obtained compatible results, considering the respective standard deviations. Therefore, the hardening processes developed by the two mixtures were effective for hardening the paste and gaining strength, either by the hydration of hydraulic compounds (BBC: binary binder concrete) or alkali-activation reactions (AABC: alkali-activated binder concrete). However, the workability of the mixtures was quite different, as shown in Figure 6. The slump of the BBC06 mixture was equal to 60 mm, while for the AABC06 mixture, it was negligible. This suspicious behavior between compressive strength and workability can be attributed to the reactions of alkali activation [136]. When mixing the EBA-SF system with the sodium hydroxide solution, the activator and precursor come into contact, and the reactions of alkali activation soon begin [68,69]. The alkali-activated binder pastes act as a glue, which prevents slipping between particles and implies low workability. In concrete with binary binder without alkali activation, the adhesion between the particles in the fresh state is lower, favoring workability. The compressive strength of the BBC05 mixture (w/b = 0.5) was 44% higher compared to the BBC06 mixture, showing compatibility between the amount of water available in the system and the water demand for reactions and formation of solid products. The balance between availability and water consumption promotes the densification of the matrix and a consequent increase in strength [137]. Therefore, the optimum point of the water/binder ratio is close to 0.5. The workability of the BBC05 mixture decreased by 20% in contrast to the BBC06 mixture (Figure 6) but remained adequate for correct casting and thickening in the molds. The same behavior did not occur with the mixture containing the alkali-activated binder. Given the negligible slump, it was necessary to increase the solution/binder ratio to promote workability. The slump of the AABC07 mixture with s/b = 0.7 was five times higher than that of the AABC06 mixture. However, the compressive strength was 12% lower. Therefore, the higher demand for water is evident in mixtures with an alkali-activated binder to make alkali-activation reactions feasible.

### 3.2. Crystallinity Peaks

**Figure 11:**
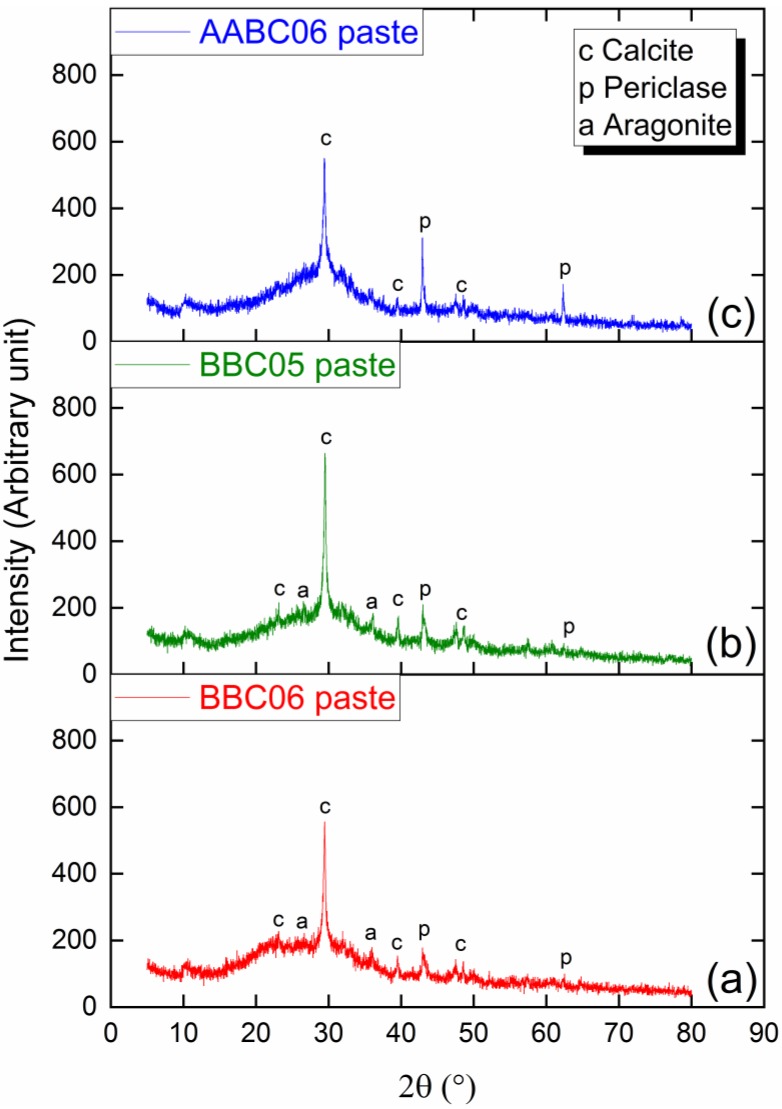
XRD of (**a**) BBC06 paste, (**b**) BBC05 paste, and (**c**) AABC06 paste.

Pastes produced from mixtures BBC06, BBC05, and AABC06 without the fine and coarse aggregates showed peaks of crystallinity (Figure 11). Some peaks of crystallinity come from raw materials, such as calcite (COD 9007689) and periclase (COD 9006456). No lime peaks were identified and, at BBC06 and BBC05, which received water, no Portlandite crystalline peaks were identified. This indicates that lime (CaO), when reacted with water did not form Portlandite (Ca(OH)_2_), probably forming calcium silicate gels through the reaction with the amorphous silica present in SF. Crystallinity peaks of aragonite (COD 9016147) were identified in the BBC06 and BBC05 pastes produced with water and without an activator. Aragonite is related to the carbonation of the C-S-H amorphous phase [138]. The peaks of calcite around 2θ = 23° and aragonite around 2θ = 28° and 2θ = 37° were not observed for the AABC06 mixture. This result indicates that the addition of activating solution promotes characteristic phase consumption of the calcite and preserves the formed C-S-H phases, preventing carbonation. In general, the binder matrix in both cases (with and without activating solution) has mostly products with amorphous phases.

### 3.3. Density, Water Absorption, and Porosity

**Figure 12:**
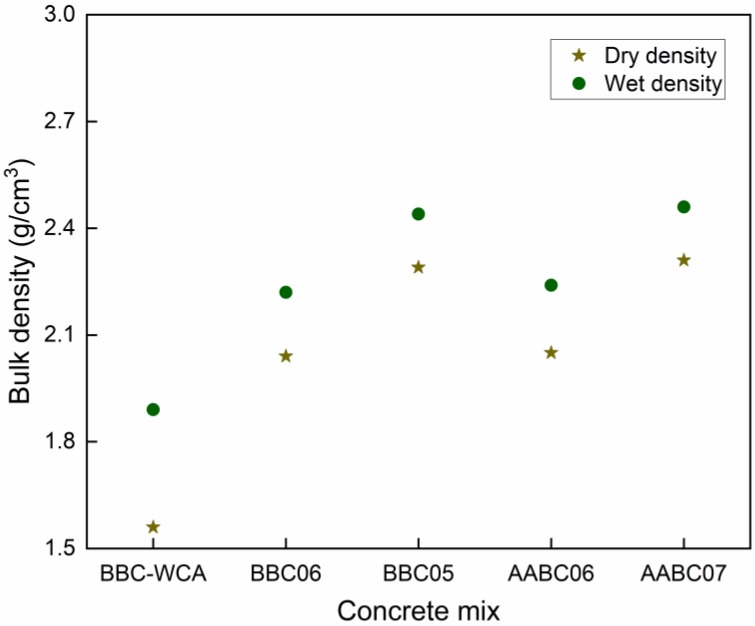
Dry density and saturated density for each concrete mix.

**Figure 13:**
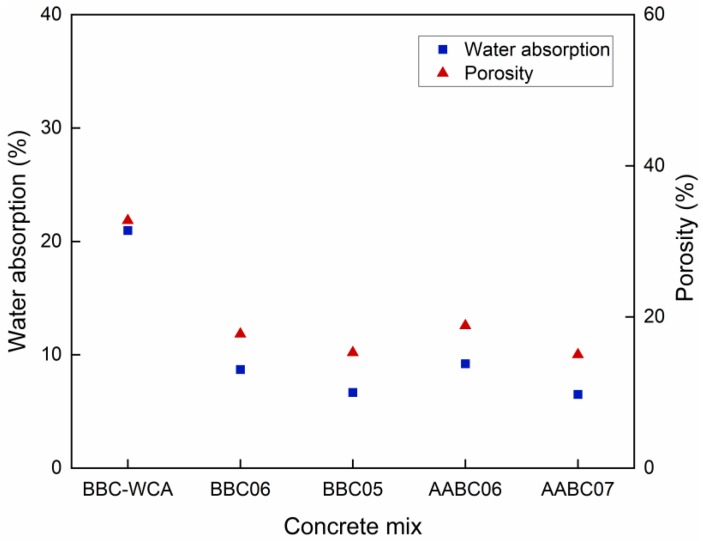
Water absorption and porosity for each concrete mix.

The results for dry density and saturated density are shown in Figure 12. In general, there was a similar trend for dry and saturated specimens. The BBC-WCA mixture obtained the lowest density, both dry and saturated (ρdry = 1.56 g/cm^3^ and ρwet = 1.89 g/cm^3^). It is noticed that the difference between dry and saturated density is greater than the other samples, which indicates the greater porosity of the material. As the pores accessible to water are filled, the density increases considerably [101]. This result is in line with the porosity and water absorption results in Figure 13. The greatest results are related to the BBC05 (ρdry = 2.29 g/cm^3^; ρwet = 2.44 g/cm^3^) and AABC07 (ρdry = 2.31 g/cm^3^; ρwet = 2.46 g/cm^3^). The BBC06 and AABC06 mixtures obtained intermediate densities. Except for the AABC07 mixture, the results observed are in line with those obtained for compressive strength—that is, the greater the compressive strength, the greater the density [89,101].

Figure 13 summarizes the results of water absorption and porosity. The BBC-WAC mixture resulted in the highest values of water absorption and porosity, which was about twice that of the others (WA = 20.97% and PR = 32.77%). The lowest values were achieved by the BBC05 (WA = 6.67% and PR = 15.28%) and AABC07 (WA = 6.50% and PR = 15.02%) mixtures. The BBC06 and AABC06 samples obtained intermediates; however, these values were close to the last two previously mentioned. The values obtained are in line with ASTM C902 [134], except for the BBC-WCA mixture. Despite measuring different physical properties, in general, water absorption and porosity resulted in similar trends. This fact refers to the nature of the properties related to the material’s permeable pores [52]. Therefore, the lower the water absorption, the lower the porosity. Likewise, both results followed the variation in dry and saturated densities; that is, the lower the water absorption and the porosity, the greater the density. Except for the AABC07 mixture, both water absorption and porosity are correlated with strength. Less water absorption and less porosity imply a greater amount of solid material in the mixture and fewer pores, which contributes to a better cohesion of the concrete constituents and a higher matrix density [49,139]. The behavior of the AABC was different and differed from that commonly used in cementitious materials. It is suggested that this behavior is attributed to the reactions of alkali activation, which promote the densification of the matrix, with the formation of a greater amount of closed pores and remaining inaccessible to water.

### 3.4. Expansibility

**Figure 14:**
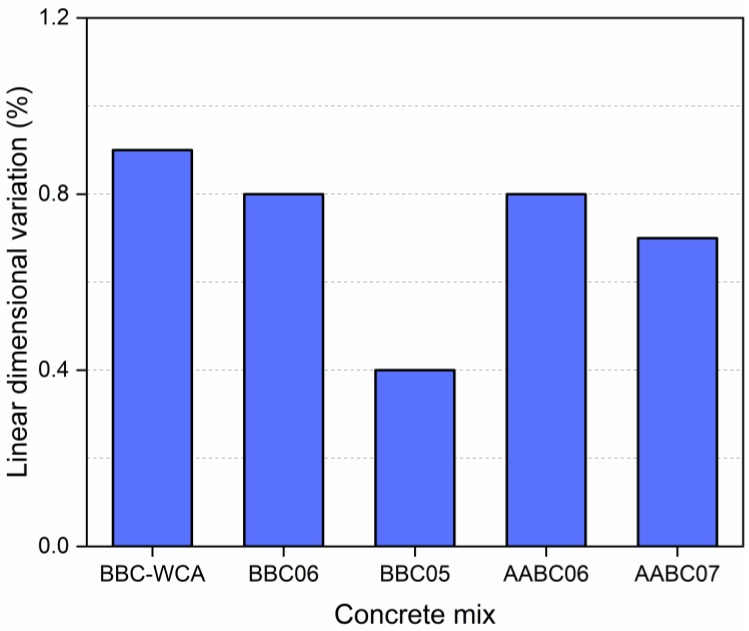
Linear dimensional variation for paver blocks produced with each concrete mix.

The expandability results are listed in Figure 14. The BBC-WCA mixture resulted in the most significant linear variation: around 0.9%. The BBC05 mixture obtained the least expandability (0.4%), while for the other mixtures, the results were intermediate, around 0.8% and 0.7%. In general, there was no significant expansion, indicating that paving blocks have good tolerance to dimensional variation after wetting and drying cycles [140]. For the BBC, the expandability followed the strength in an inversely proportional way; therefore, the higher the strength, the lower the expandability. The densification of the binder matrix can explain this behavior. Therefore, less expandability corresponds to greater cohesion between the constituents of concrete, higher strength, less porosity, less water absorption, and greater density. The AABC, in turn, presented an inverse behavior; that is, the expandability followed the strength directly. The products generated by alkali activation, although denser and less porous, are less strength, so there was a reduction in expandability. 

### 3.5. Abrasion Resistance

**Figure 15:**
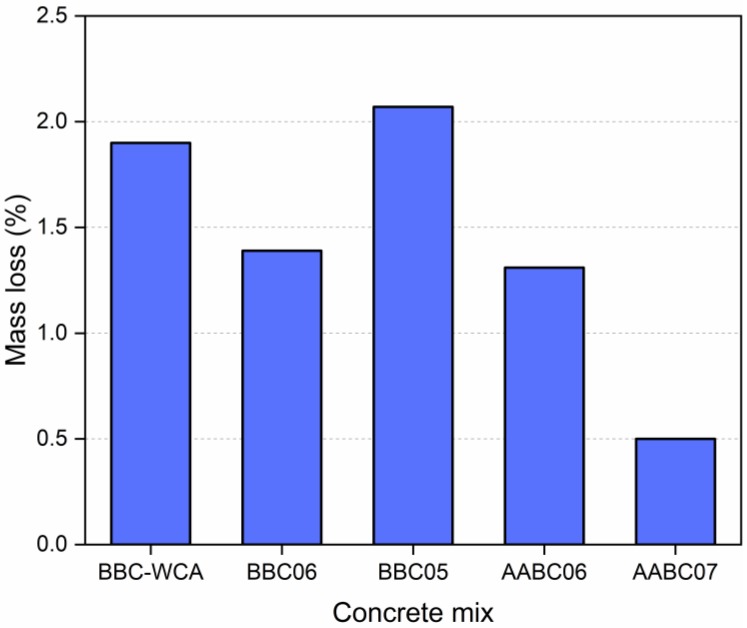
Mass loss after in-depth abrasion test for each mixture.

The results of the deep abrasion test are shown in Figure 15. In general, the results contrast with those obtained in the other properties evaluated. The BBC05 mixture obtained the most significant loss of mass (2.07%), despite corresponding to the highest strength, the highest densities, and the lowest porosities. The weight loss of the BBC-WCA mixture was around 1.90%. The BBC06 and AABC06 mixtures result in equivalent mass losses, 1.39% and 1.31%, respectively. The lower mass loss was attributed to the AAB07 mixture, which has a high density and low porosity compared to the universe of samples under analysis; however, the compressive strength was not as high. Therefore, the resistance to frictional surface wear is more related to the material structure, surface conditions, and matrix proportions [141]. Alkali activation reactions resulted in solid products that are more resistant to wear, depending on the water content, in addition to intensifying the adhesion between aggregates and binder paste [92].

### 3.6. Potential for Degradation of Organic Matter

**Figure 16:**
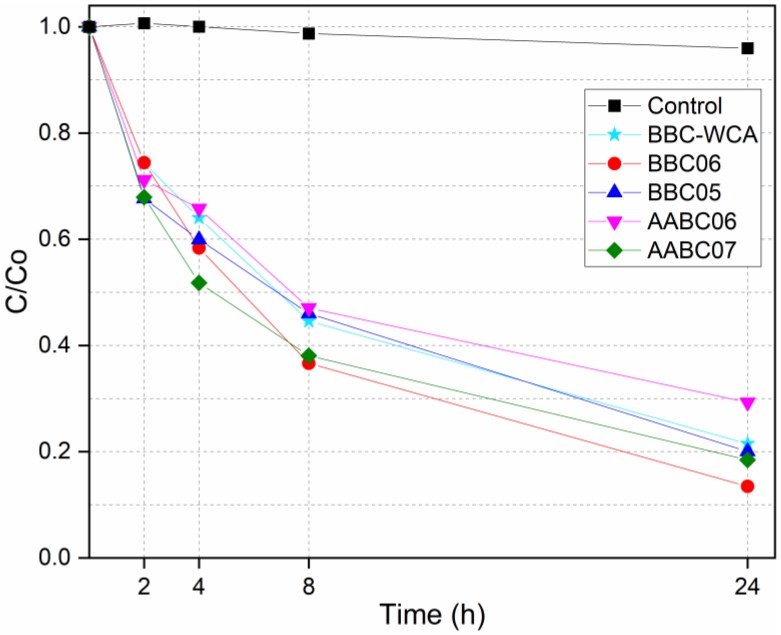
Dye concentration versus time in the dark.

**Figure 17:**
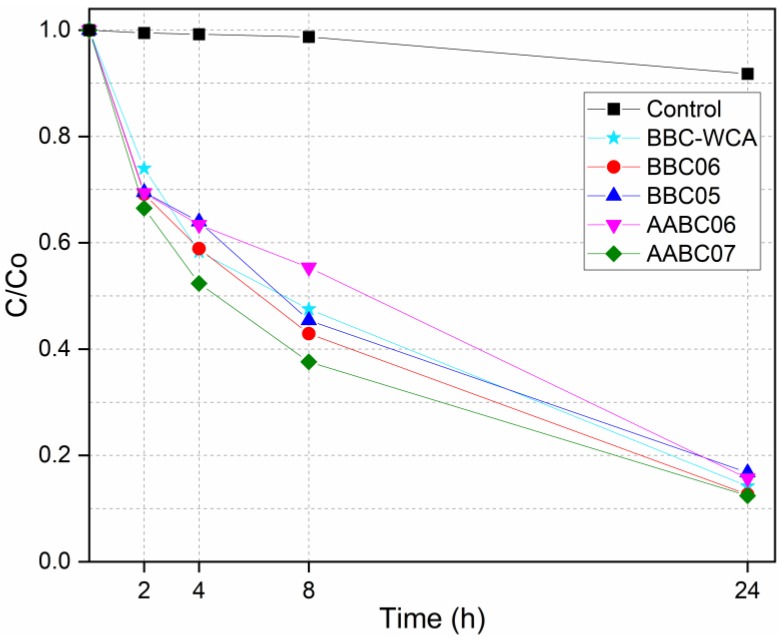
Dye concentration versus time under UV light.

**Figure 18:**
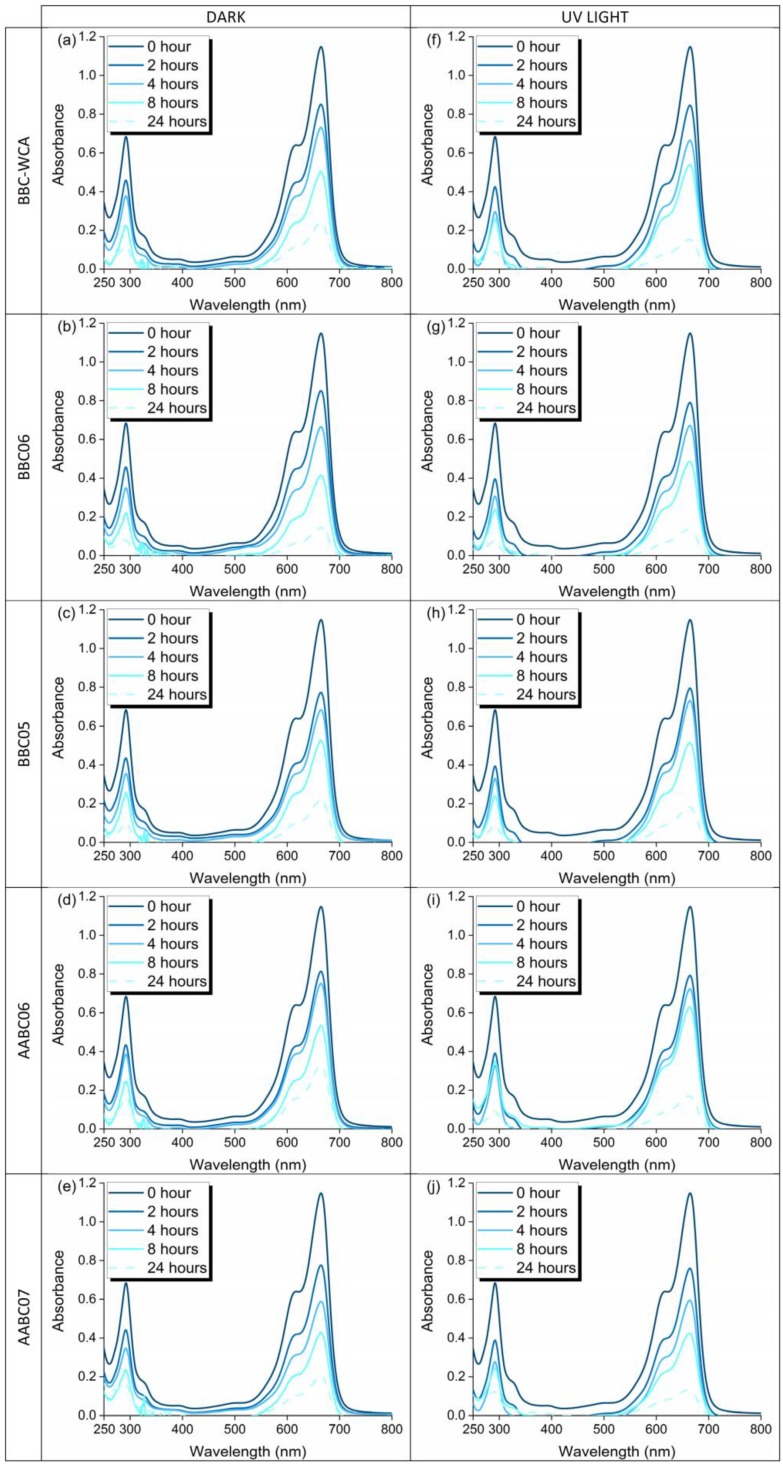
Absorbance curves for each mixture (**a**–**e**) in the dark and (**f**–**j**) under UV light.

The relative concentrations of the methylene blue solution as a function of the exposure time to the samples of EBA-SF binder paste, in the dark and under UV light, are listed in Figure 16 and Figure 17, respectively. Figure 18 shows the absorbance curves for each mixture, in the dark and under UV light. Each absorbance curve corresponds to a concentration, which was calculated using the calibration equation defined and presented in the topic *Materials and Methods*. The concentration of the reference solution was defined as C_0_, while the solution analyzed after contact with the material was called C. The C/C_0_ ratio identifies the relative concentration. The self-cleaning properties are closely related to the material’s ability to function as an active site for the degradation of organic matter.

In general, there was a similar trend of organic matter degradation for all samples analyzed, both in the dark and under UV light. In the dark, a greater dispersion of results can be seen in 4 h and especially in 24 h. The highest concentrations were obtained by the samples in contact with the AABC06 mixture and the lowest referring to the BBC06 mixture. Under UV light, concentrations differ within 8 h of exposure but converge to a common point within 24 h. The highest concentrations are related to the AABC06 mixture and the lowest are related to the AABC07 mixture. The relative concentrations can be divided into three intervals, according to the mixtures, both for dark and under UV light. The first concerns the BBC06 and AABC07 mixtures, which resulted in the lowest concentrations and consequently a more significant degradation of methylene blue. In the second interval are the BBC05 and BBC-WCA mixtures, which resulted in intermediate concentrations. The AABC06 mixture, alone, integrates the third interval, in which there were the highest concentrations and the least degradation of organic dye.

Both studied binders (binary binder, BB; and alkali-activated binder, AAB) were able to reduce the concentration of methylene blue present in the solutions, both in the dark and under UV light. This result can be explained by three phenomena: (i) adsorption, (ii) leaching, and (iii) degradation by photocatalysis. The pore network of the binder paste functioned as a site for the adsorption of methylene blue. Thus, the dye migrated from the solution to the surface of the pore network of the binder, remaining adsorbed and decreasing the concentration of the solution. In conjunction with adsorption, some solid phases present in the EBA-SF system leached into the solution, increasing the porosity and surface area, which intensified the methylene blue adsorption. On the other hand, samples submitted to ultraviolet radiation showed a more significant degradation of methylene blue, especially in 24 h, in contrast to samples in the dark, which indicates photocatalytic activity, in addition to adsorption and leaching. Besides, the convergence in the concentration values under UV light in 24 h showed that the type of mixture is not very influential; that is, all the mixtures obtained meaningful results in terms of dye degradation.

### 3.7. Microstructural Analysis

**Figure 19:**
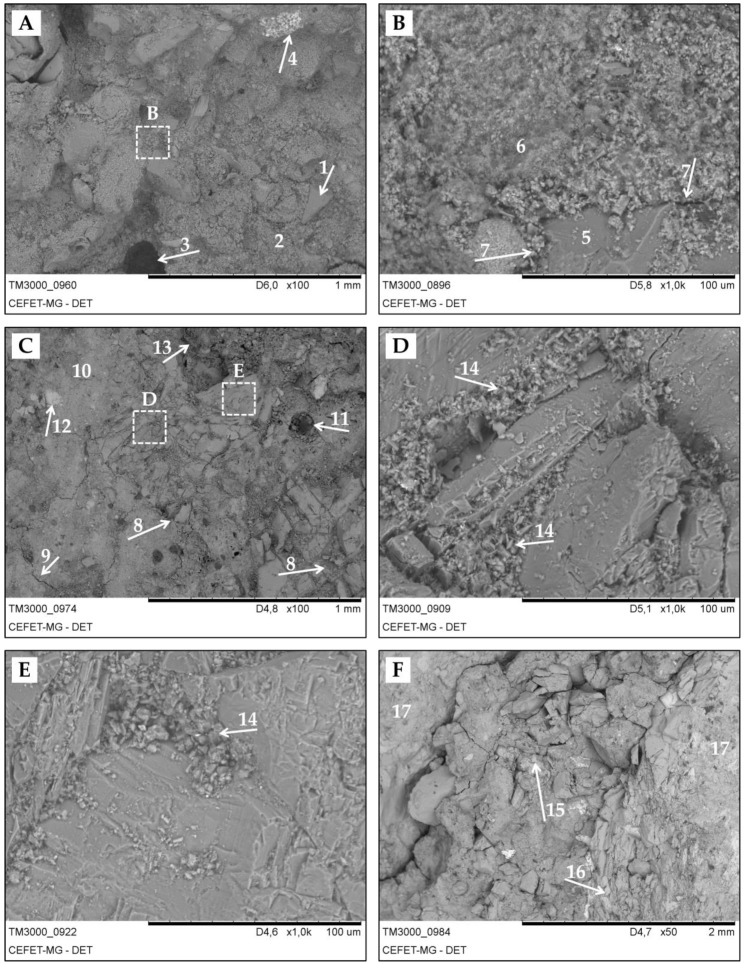
SEM images of binary binder concrete. (**A**) BBC-WCA–100X; (**B**) BBC-WCA–1000X; (**C**) BBC06–100X; (**D**) BBC06–1000X–1; (**E**) BBC06–1000X–2; (**F**) BBC06–50X; et al.

**Figure 20:**
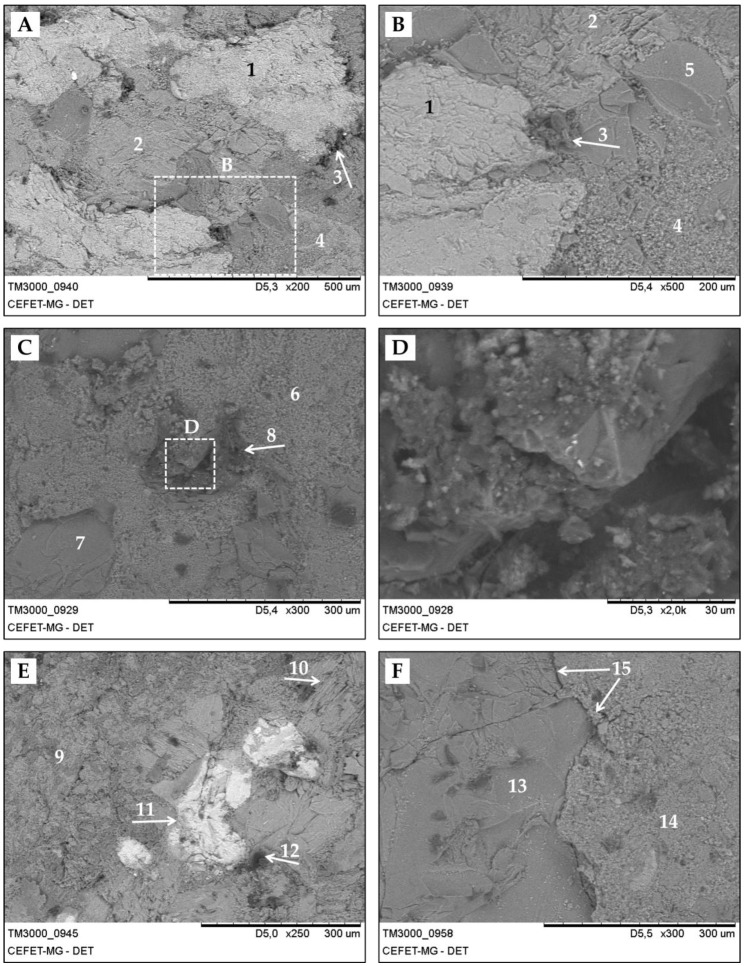
SEM images of alkali-activated binder concrete (**A**) AABC-06–200X; (**B**) AABC-06–500X; (**C**) AABC-06–300X; (**D**) AABC-06–2000X; (**E**) AABC-06–250X; (**F**) AABC-06–250X;.

Figure 19 and Figure 20 depict images obtained by SEM for samples of binary binder concrete and alkali-activated binder concrete, respectively. Figure 19A shows the surface of a BBC-WCA sample. Aggregates were identified—stone powder—of the order of 0.2 mm (detail 1) with an angular aspect, a well-finished and smooth surface, immersed in a cementitious matrix (detail 2) with a rough appearance. We also noticed the presence of pores (detail 3) and denser phases in the matrix (detail 4) identified by the lighter color. As the images were obtained using a backscattered electron detector, the denser material corresponds to the lighter tones, and the less dense material corresponds to the darker colors [142]. Figure 19B shows an enlargement of the previous figure, where it is possible to notice the aggregate (detail 5), the binder matrix (detail 6), and the interfacial transition zone (ITZ, detail 7). The rough/porous aspect identifies a less cohesive matrix, which corroborates the results of strength, porosity, and density. In turn, Figure 19C shows a sample from BBC06, where fractured aggregates (detail 8) and microfissures (detail 9) were identified, which were caused by the mechanism of sample extraction. However, the cementitious matrix (detail 10) has a much denser and cohesive appearance compared to the BBC-WCA line, confirming the results of greater strength, lower density, and less porosity. Pores (detail 11), regions with more dense material (detail 12), and regions with less dense material (detail 13) were observed. Figure 19D,E correspond to the enlargement of Figure 19C at the indicated points, where it is possible to observe a fragmented structure (detail 14), initially of cohesive material, covered by loose lamellar particles. It is probable that the particles got loose from the cohesive material and filled the spaces around it. Figure 19F represents the lowest magnification studied, where the cementitious matrix is fragmented into larger portions in the center (detail 15), while in the lower right corner, the matrix cracked and fractionated into smaller pieces, following a vertical trend (detail 16). More to the right and the left, the matrix remains intact (details 17).

SEM images for the alkali-activated binder concrete (Figure 20) are peculiar and showed significant differences with the binary binder concrete. The marked difference refers to the presence of distinct phases, which are observed throughout the extension of the alkali-activated matrix (Figure 20A,B,E). These phases are identified by lighter and darker tones, which are associated with the density of the phase formed with the reactions of alkali activation. Using this methodology, four phases are visually identified, indicated by details 1, 2, 3, and 4, which can also be seen in Figure 20B, which is an enlargement of the previous figure. The lighter phase (detail 1), and consequently the denser phase, has a cohesive texture and appears to have lamellar structures on the surface. The intermediate phase, in terms of density and intensity of contrast, was very similar to the denser phase; however, it appears to be more cohesive. The darker and less dense phase (detail 3) showed a less cohesive structure with convex contour components. Another region was also identified (detail 4) that was much less cohesive than the others, appearing to have granular material adhered to the surface. In addition, in Figure 20B, it is also possible to notice the presence of aggregates—filler—of the order of 100 µm (detail 5). Figure 20C identifies another region of the alkali-activated binder concrete, and it was possible to observe a main continuous phase (detail 6) with a coarser aspect. Detail 7 identifies an aggregate of the order of 200 µm, which is certainly a filler originating from stone powder. The darker and less dense phase (detail 8) appears to be less cohesive and resembles the phase of detail 3. Figure 20D shows an enlargement of Figure 20C in the region of the darkest phase to further investigate this area. At first, these phases have little cohesion; the presence of smaller particles adhered to the larger ones and empty spaces. A similar phase is also present in binary binder concrete, however, there is less occurrence. Possibly, this darker and less dense phase is some remaining mineral or vegetable phase of the EBA that did not react during the process of alkali activation. Figure 20E shows another region of the AABC, where five phases are identified (details 9, 10, 11, 12, and 13). The first phase mentioned (detail 9) represents a continuous but disordered structure with a coarse aspect. The second phase (detail 10) provides the same contrast as the first phase; however, it has ordered lamellar structures. The detail 11 phase also results in lighter shades and has a more cohesive structure, which justifies its contrast, which can be associated with a higher density compared to the other phases. Again, the darker phase (detail 12) appears punctually. Figure 20F shows the interaction between aggregate and matrix, where it is possible to observe an aggregate (detail 13) immersed in the matrix of alkali-activated binder (detail 14) and, in the interface between the phases, highlight the ITZ (detail 15).

## 4. Conclusions

This work carried out a comprehensive study on low-carbon concrete based on industrial waste agglomerate to produce ecological paving blocks. Given the results achieved, the following conclusions can be highlighted:

1. All mixtures achieved significant compressive strengths, identifying the cementing and bonding capacity of the EBA-SF waste system. The greatest strength was achieved by the BBC06 mixture, in which the water content was optimized. Thus, demand and availability were compatible with water for the formation of solid products. The BBC-WCA trait is related to a lower strength.

2. Mixtures with binary binder without alkali activation showed significant workability, unlike mixtures with the alkali-activated binder, for which there was little workability.

3. The structure investigation of the binder pastes by means of XRD identified peaks of crystalline phases of calcite and periclase, which were related for raw materials, and aragonite, which was a result of the C-S-H carbonatation. Lime and Portlandite peaks ware not identified, corresponding to the reaction between lime from EBA and amorphous silica from SF.

4. Dry density tended to be similar to saturated density. Mixtures with a higher density are those with a denser binder matrix, and a greater number of solid products were formed during stiffening and strength gain.

5. Filling spaces between aggregate grains was effective for both the BBC and the AABC. As the water content was optimized, the voids were filled with solid products resulting from the hardening of the EBA-SF system.

6. The expandability is more accentuated in the AABC, indicating that the products formed by the alkali-activation reactions are more susceptible to linear dimensional variation in the presence of water. BBC05 resulted in the lowest expandability values.

7. The BBC is more vulnerable to surface wear compared to the AABC. Abrasion resistance is more related to the binder used than to the compressive strength.

8. The photocatalytic potential was confirmed by the degradation of more pronounced organic matter under UV light. In addition to photocatalysis, adsorption also occurs in the pore network of the binder paste and phase leaching of the EBA-SF system.

9. SEM images contributed to evaluating aspects such as the morphology, surface, and particle arrangement present in mixtures at the microstructural level.

10. The paving blocks produced with the BBC05 dosage presented the minimum requirements requested by standards for paving blocks produced from Portland cement concrete. The positive aspects are the full use of waste to produce the binder and the non-use of alkaline activators.

Thus, the potential for using the EBA-SF waste system as a low-carbon binder to produce ecological paving blocks is evident. In addition to achieving adequate performance in terms of strength, water absorption, porosity, expandability, and wear, the new material evaluated collaborates to mitigate impacts on the environment and boost sustainable development.

## Figures and Tables

**Table 1:** Chemical composition of EBA and silica fume (SF).

	CaO	SiO_2_	Al_2_O_3_	Fe_2_O_3_	MgO	K_2_O	P_2_O_5_	TiO_2_	Na_2_O	LOI ^a^
EBA	44.71	1.23	7.03	2.37	5.59	4.12	2.97	1.46	0.28	30.22
SF	0.75	95.41	0.51	0.09	0.07	2.79	0	0	0	0.34

Notes: ^a^ LOI—Loss on ignition at 1000 °C.

**Table 2:** Materials proportion design.

Mix	Proportion (%)									
	EBA ^a^	Silica Fume	Sand	Stone Dust	C. Ag. ^b^ [4.8–9.5] ^c^	C. Ag. ^b^ [9.5–19.5] ^c^	Water	NaOH Solution	Superp. ^d^	w/b or s/b Ratio ^e^
BBC-WCA	13.1	13.1	26.2	26.2	-	-	21.0	-	0.26	0.8
BBC06	8.9	8.9	17.8	17.8	17.8	17.8	10.7	-	0.36	0.6
BBC05	9.1	9.1	18.1	18.1	18.1	18.1	9.1	-	0.36	0.5
AABC06	8.9	8.9	17.8	17.8	17.8	17.8	-	10.7	0.36	0.6
AABC07	8.7	8.7	17.5	17.5	17.5	17.5	-	12.2	0.36	0.7

Notes: ^a^ EBA: Eucalyptus biomass ash; ^b^ Coarse aggregate; ^c^ Coarse aggregate, dimensions in millimetres; ^d^ Superplasticizer; ^e^ Water/binder ratio (w/b) or solution/binder ratio (s/b).
